# Microbial-generated amyloids and Alzheimer's disease (AD)

**DOI:** 10.3389/fnagi.2015.00009

**Published:** 2015-02-10

**Authors:** James M. Hill, Walter J. Lukiw

**Affiliations:** ^1^Louisiana State University Neuroscience Center, Louisiana State University Health Sciences CenterNew Orleans, LA, USA; ^2^Departments of Ophthalmology, Louisiana State University Health Sciences CenterNew Orleans, LA, USA; ^3^Microbiology, Louisiana State University Health Sciences CenterNew Orleans, LA, USA; ^4^Pharmacology, Louisiana State University Health Sciences CenterNew Orleans, LA, USA; ^5^Neurology, Louisiana State University Health Sciences CenterNew Orleans, LA, USA

**Keywords:** 42 amino acid amyloid-beta peptide (Aβ42), Alzheimer's disease (AD), birefringence, central nervous system (CNS), Congo red, innate immunity, microbiome, toll receptor type 2 (TLR2)

## Introduction

Atypical amyloid generation, folding, aggregation and impaired clearance are characteristic pathological features of human neurodegenerative disorders including Alzheimer's disease (AD). What is generally not appreciated is that a major secretory product of microbes is amyloid, and that the contribution of microbial amyloid to the pathophysiology of the human central nervous system (CNS) is potentially substantial. While earlier findings suggested that these amyloids may serve some immune-evasive strategy, it has recently become evident that humans have a tremendously heavy systemic burden of amyloid which may contribute to the pathology of progressive neurological diseases with an amyloidogenic component. This perspective will highlight some recent inroads made into our understanding of the enigmatic role that microbial amyloids may play in the homeostasis and pathology of the CNS with particular reference to AD wherever possible.

## Amyloid: microbial and CNS sources

“*Amyloid*” is a generic term for any aggregated, insoluble, lipoprotein-rich deposit exhibiting β-pleated sheet structures oriented perpendicular to the fibrillar axis (Steensma, 2001; Badtke et al., 2009; Blanco et al., 2012; Buxbaum and Linke, 2012). Amyloids are characterized by an apple-green birefringence (λ_max_ ~555 nm) when stained with the secondary diazo-dye Congo-red (λ_max_ ~498 nm) when viewed under polarized light (upon binding to amyloid, Congo Red displays bright fluorescence emission at λ_max_ ~614 nm after excitation at λ_max_ ~497 nm; Alexandrov et al., 2011; O'Brien and Wong, 2011). Amyloid fibrillation is initiated by self-aggregation of protein monomers-into-dimers, oligomers and fibrils, which accumulate over time, and this process is thought to result from the hydrophobic nature of the aromatic amino-acid peptides comprising the primary sequence of the amyloid (O'Brien and Wong, 2011; Lukiw, 2012). The Congo red dye-based intercalation of β-pleated sheets, induction of a positive anisotropy that is polarized and directionally dependent, and generation of a measureable wavelength shift and apple-green birefringence is the hallmark of all amyloids and is the “gold standard” in the diagnosis of amyloidogenic disease (Linke, 2006; Buxbaum and Linke, 2012). The polymerization of amyloidogenic proteins is cooperative, and can be accelerated by amyloid aggregates derived from the same protein in a selective “seeding” process. The identification of the “amylome,” a classification of amino acid sequences within proteins with internal, self-complementary interfaces and high fiber-forming propensity has improved our understanding of the capability of different proteins to form amyloids that contribute to “dense-deposit” disease (Goldschmidt et al., 2010; O'Brien and Wong, 2011; Lukiw, 2012). The pathogenesis of diseases that accumulate amyloid, including AD, all involve a marked inflammatory response at sites of amyloid deposition, and this is mediated by microglial cells, the “roving macrophages” of the CNS. Microglia appear to utilize molecular sensors on their external surface, such as the Toll-like receptor 2, TLR2, to recognize abnormal forms of amyloid and initiate a phagocytic or “clearance” response (Zhao et al., 2013; Ferrera et al., 2014; Jones et al., 2014). Here we describe a relatively recent collection of stimulating research at the crossroads of microbial and AD amyloids highlighting 5 recent, specific and illustrative insights into the potential contribution of microbial-derived amyloids to CNS amyloidogenesis and AD.

### Microbiome-derived amyloids

The microbiome is the aggregate of all microorganisms that reside on and within the human body, forming a complex ecosystem that includes the skin, oral and nasal mucosa, the urogenital and gastrointestinal (GI) tracts. The microbiome of the GI tract is by far the largest reservoir of microbes in the human body, containing about 10^14^ microbes; over 99% of microbiota in the GI tract are anaerobic bacteria, with fungi, protozoa, archaebacteria and other microorganisms making up the remainder (Bhattacharjee and Lukiw, 2013; Hill et al., 2014; Lin et al., 2014). Prokaryotic cells of the human microbiome outnumber human eukaryotic host cells by about 100 to 1, and collectively microbial genes outnumber host genes by about 150 to 1 (Hill et al., 2014; Lin et al., 2014). Recent microbiome analysis has revealed that the majority (98%) of all GI tract species belongs to only 4 major bacterial divisions: the gram-positive *Firmicutes* (64%) and *Actinobacteria* (3%) and the gram-negative *Bacteroidetes* (23%) and *Proteobacteria* (8%). The remaining 2% consist of minor taxonomic divisions which are quite diverse (Hattori and Taylor, 2009; Bhattacharjee and Lukiw, 2013; Schwartz and Boles, 2013). Many different microbiome species including fungi and bacteria secrete amyloid (Hill et al., 2014; Syed and Boles, 2014). For example, amyloids are associated with fungal surface-structures and the recent observation of amyloidogenic fungal proteins and diffuse mycoses in the blood of AD patients suggest that chronic fungal infection increases AD risk (Alonso et al., 2014a,b; Hill et al., 2014). To cite one other recent example, in *Escherichia coli* extracellular amyloids known as curli fibers, composed of the major structural subunit CsgA, are a common secretory component that facilitate surface attachment and adhesion, biofilm development and protection against host defenses (Schwartz and Boles, 2013; Asti and Gioglio, 2014). Biofilms represent a matrix of extracellular polymeric amyloids and other lipoproteins in various structural forms. Interestingly, the extracellular 17.7 kDa CsgA amyloid precursor contains a pathogen-associated molecular pattern (PAMP) that, like the Aβ42 peptide, is recognized by the human immune system TLR2 (Zhou et al., 2012). An expanding list of bacterial amyloid systems include those associated with gram-negative species of *Streptomyces, Bacillus, Pseudomonas, Staphylococcus* and others, suggesting that functional amyloids are a widespread phenomenon utilized by a wide diversity of microbiome-bacteria (Schwartz and Boles, 2013; Asti and Gioglio, 2014; Hill et al., 2014). Indeed the extremely large number and variety of microbiome bacteria and their capability to produce vast quantities of amyloids indicates that human physiology may be potentially exposed to a tremendous systemic amyloid burden, especially during aging when the GI tract epithelial and blood-brain barriers become significantly more restructured and permeable (Bhattacharjee and Lukiw, 2013; Marques et al., 2013; Oakley and Tharakan, 2014).

### The amyloid peptides of AD and endotoxin-mediated inflammation

A ~770 amino acid type 3 transmembrane β-amyloid precursor protein (βAPP), through interaction with membrane proteins and tandem secretase cleavage yields a series of ragged Aβpeptide monomers, two of the most abundant being the 40 and 42 amino acid peptides Aβ40 and Aβ42 (Van Broeck et al., 2007; O'Brien and Wong, 2011; Zhang et al., 2012). Aβ40 peptides associate with the endothelium of the cerebral vasculature, and the more neurotoxic, albeit less abundant, hydrophobic Aβ42 species form the nuclei of the senile plaque (SP) lesions of AD (Alexandrov et al., 2011; O'Brien and Wong, 2011). Interestingly, the extra two hydrophobic amino acids in Aβ42 (vs. Aβ40) appear to convey many of the toxic biophysical attributes and self-aggregation of this slightly larger molecule (Zhou et al., 2011; Teng et al., 2012; Zhang et al., 2012). The recognition of Aβ42 peptides and their misfolded aggregates by microglial-surveillance systems, and the inability of microglial cells to deal with these toxic, pro-inflammatory inclusions are thought to form the molecular basis for the elevated oxidative stress, aberrant immune-activation and chronic inflammation characteristic of AD brain (Armstrong, 2006; Cui et al., 2007; Van Broeck et al., 2007; Boutajangout and Wisniewski, 2013; Furukawa and Nukina, 2013; Ferrera et al., 2014; Serpente et al., 2014; Takeda et al., 2014). Interestingly (i) βAPP-derived Aβ42 peptides induce a pattern of expression of inflammatory genes typical of the classical immune- and inflammatory-response induced by infectious agents such as bacterial lipopolysaccharide (LPS), a common endotoxin of the outer membrane of gram-negative bacteria (Colangelo et al., 2002; Ferrera et al., 2014), and (ii) the presence of bacteria, LPS or endotoxin-mediated inflammation strongly contributes to amyloid neurotoxicity (Hammer et al., 2008; Dasari et al., 2011; Blanco et al., 2012; Zhou et al., 2012; Serpente et al., 2014).

### Congo red staining to identify amyloid deposits

Congo red [3,3′-([1,1′-biphenyl]-4,4′-diyl)bis(4-aminonaphthalene-1-sulfonic-acid-disodium salt)], a toxic, water soluble secondary diazo dye, was first synthesized in 1883 and used as a textile dye (Steensma, 2001; Linke, 2006). Due to its toxicity, Congo red's use in textile dying was discontinued, but because of its important spectroscopic properties gained wide use in investigative microbiology (Linke, 2006). The proposed Congo red-mediated staining mechanism suggests a substrate-mediated hydrophobic pi-pi orbital stacking interaction between the aromatic rings of the dye molecules and β-pleated sheet structures of both amyloids and textiles (Buxbaum and Linke, 2012). Congo red was classically used in microbiological epidemiology as a bacterial-stain, for example, to rapidly identify the presence of virulent forms of gram-negative *Shigella* where the dye binds the bacterium's unique LPS surface structures (Linke, 2006; Buxbaum and Linke, 2012). Congo red's apple-green birefringent fluorescence under polarized light when bound to amyloid fibrils is currently used as a sensitive diagnostic tool for amyloidosis including the Aβ42-enriched SPs of AD (Steensma, 2001; Linke, 2006; Buxbaum and Linke, 2012). Interestingly it has very recently been found that (i) LPS is capable of inducing a pathogenic Congo red-sensitive β-pleated sheet conformation in prion amyloids (Saleem et al., 2014); and (ii) the infectious microbial burden is significantly associated with both AD development and the propensity of AD amyloids to be stained by Congo red (Bu et al., 2014).

### Molecular mimicry between mitochondria and bacteria

Because mitochondria appear to have originated from bacteria via endosymbiotic relationships that formed very early in the evolutionary history of eukaryotes, cross-reactivity of mitochondria and immunological responses to bacterial amyloid or LPS may have deleterious effects on mitochondrial function. This “molecular mimicry” is partially evidenced by extragastric diseases such as the basal ganglia disorder Sydenham's chorea, rheumatic fever, low-grade systemic-inflammatory states and the link to the *Firmicute Streptococcus* and/or the gram-negative microaerophilic *Proteobacteria Helicobacter pylori* (Douglas-Escobar et al., 2013; Hayashi, 2013; Hornig, 2013; Roubaud Baudron et al., 2013; Hill et al., 2014). Previous bacterial infection resulting in antibody formation to amyloids or bacterial endotoxins may predispose CNS mitochondria or amyloids to subsequent attack by antibodies resulting in the up-regulation of inflammatory signaling (Bu et al., 2014).

### Activation of TLR2 by amyloids

TLRs are type I membrane-spanning protein receptors expressed in microglial cells. TLRs play key roles in host protection from microbial-invasion via the activation of the innate-immune system by sensing structurally conserved PAMPs from microbes that are distinguishable from, and not innate to, the host organism (Tükel et al., 2009; Harry, 2013; Yu and Ye, 2014). Of the 13 currently identified TLRs (TLR1 to TLR13) the microglial TLR2s are activated by amyloid, lipoproteins and other microbial triggers that subsequently induce cytokine production, inflammation, phagocytosis and innate immune defense responses that directly impact CNS homoeostasis and drive neuropathology. More specifically the TLR2/TLR1 complex can recognize biofilm-associated amyloids produced by *Firmicutes, Bacteroidetes*, and *Proteobacteria* (Nishimori et al., 2012; Bhattacharjee and Lukiw, 2013; Asti and Gioglio, 2014). Remarkably, the Aβ42 peptides of AD that are associated with robust microglia-mediated inflammatory responses also activate TLR2 (Gustot et al., 2013; Yu and Ye, 2014). Of further interest is (i) that microbial amyloids induce pro-inflammatory interleukin IL-17A, a driver of NF-kB signaling and cyclooxygenase-2 activation, and other potent mediators of inflammatory-responses such as IL-22 via direct TLR2 activation (Nishimori et al., 2012); and (ii) that increased levels of IL-17A and IL-22 are associated with chronic inflammatory diseases including AD (Zhang et al., 2013).

## Concluding remarks

Diverse microbes of the human microbiome generate functional amyloids. Their ability to bind Congo red has provided useful tools for characterizing both microbial- and CNS-derived amyloids (Zhou et al., 2012; Serpente et al., 2014). The large amount of microbial-generated GI amyloid implicates high potential systemic exposure to bacterial amyloid, and the bioavailability of amyloid to the CNS increases as humans age (Bhattacharjee and Lukiw, 2013; Marques et al., 2013; Tran and Greenwood-Van Meerveld, 2013; Oakley and Tharakan, 2014). Microbial and CNS amyloids are biologically similar in their structure and immunogenicity and complex mechanistic interrelationships between these amyloids are beginning to emerge. It is remarkable **(i)** that human microbes that produce amyloids such as CsgA and curli, and the Aβ42 peptides that accumulate in AD, are recognized by the same TLR2/TLR1 immune sensor-receptor system of the 13 different TLR-type receptors available; and **(ii)** that they direct the same up-regulation of IL-17A- and IL-22-mediated pro-inflammatory-signaling (Rapsinski et al., 2013; Zhang et al., 2013; Yu and Ye, 2014). Interestingly, CsgA and Aβ42 peptides do not share common amino-acid sequences, only structural similarity in their PAMPs. Microbes or their secretory or degradation products including their amyloids and LPSs are powerful inflammatory activators and inducers of cytokines and complement proteins, affecting vascular permeability and generating free-radicals that further support amyloidogenesis (Hill et al., 2014; Lin et al., 2014). These pathogenic signaling features are also highly characteristic of AD neuropathology. A more detailed understanding of human microbial ecosystems and their amyloids should give insight into amyloid-misfolding and their contribution to inflammatory-signaling in health, aging and disease. It will certainly be interesting to see: **(i)** if any microbial-generated amyloids co-localize with the amyloid-dense SP deposits of AD; **(ii)** if GI tract microbiome-derived amyloids become more available systemically as humans age; and **(iii)** what the evolution and nature of amyloid-related communication between the GI-tract and the CNS has on the development or propagation of amyloidogenesis in pro-inflammatory degenerative disease.

### Conflict of interest statement

The authors declare that the research was conducted in the absence of any commercial or financial relationships that could be construed as a potential conflict of interest.
